# Exercise-induced neuroplasticity: a new perspective on rehabilitation for chronic low back pain

**DOI:** 10.3389/fnmol.2024.1407445

**Published:** 2024-06-07

**Authors:** Jianpeng Zou, Shijie Hao

**Affiliations:** ^1^Department of Rehabilitation and Physiotherapy, Affiliated Hospital of Shandong University of Traditional Chinese Medicine, Jinan, China; ^2^College of Rehabilitation Medicine, Shandong University of Traditional Chinese Medicine, Jinan, China

**Keywords:** exercise-induced neuroplasticity, chronic low back pain, central mechanism, psychosomatic dysfunction, exercise

## Abstract

Chronic low back pain patients often experience recurrent episodes due to various peripheral and central factors, leading to physical and mental impairments, affecting their daily life and work, and increasing the healthcare burden. With the continuous advancement of neuropathological research, changes in brain structure and function in chronic low back pain patients have been revealed. Neuroplasticity is an important mechanism of self-regulation in the brain and plays a key role in neural injury repair. Targeting neuroplasticity and regulating the central nervous system to improve functional impairments has become a research focus in rehabilitation medicine. Recent studies have shown that exercise can have beneficial effects on the body, such as improving cognition, combating depression, and enhancing athletic performance. Exercise-induced neuroplasticity may be a potential mechanism through which exercise affects the brain. This article systematically introduces the theory of exercise-induced neuroplasticity, explores the central effects mechanism of exercise on patients with chronic low back pain, and further looks forward to new directions in targeted neuroplasticity-based rehabilitation treatment for chronic low back pain.

## 1 Introduction

Chronic low back pain (CLBP) can have a certain degree of impact on the patient’s physical structure and function, daily activities, and social participation. In the modern medical model, CLBP pain is considered as a biopsychosocial syndrome. Functional impairments in CLBP pain patients involve three levels: biologically, it is mainly manifested as local pain, limited function, and reduced ability to perform daily activities; psychologically, it is manifested as fear of movement, catastrophizing thoughts about pain, anxiety, depression, and reduced self-efficacy; socially, it is manifested as impaired social functioning, work absenteeism, and overall reduction in quality of life (Tagliaferri et al., 2020). Behind these various functional impairments, changes in brain structure and function may be one of the important pathological mechanisms. Based on this, the rehabilitation goals for CLBP have also changed, shifting from excessive focus on pain management and improvement of back symptoms to targeting the central nervous system to improve the overall physical and mental functional impairments of patients. In recent years, people have become increasingly aware of the appearance of a series of functional impairments and the prolonged course of CLBP, which are closely related to neuroplasticity changes (Nijs et al., 2017). Therefore, inducing neuroplasticity changes through certain therapies may be a feasible measure to improve the overall rehabilitation effectiveness of CLBP. Exercise, as a traditional rehabilitation therapy, plays an important role in inducing neuroplasticity and promoting disease recovery (Johansson et al., 2020). Exercise has characteristics such as planning and organization, and it is a collection of repetitive physical activities aimed at maintaining or promoting physical health. Its impact on brain structure and function has attracted increasing attention. Based on the current relevant research, this paper firstly introduces in detail the theory and molecular mechanism of exercise-induced neuroplasticity, secondly proposes a new concept of exercise-targeted neuroplasticity for the treatment of CLBP and outlines its mechanism, and lastly puts forward the concept of central-peripheral rehabilitation treatment for CLBP and looks forward to the prospect of applying mind–body exercise in the rehabilitation treatment of CLBP.

## 2 Exercise-induced neuroplasticity

Neuronal plasticity is the ability of the nervous system to respond to internal and external stimuli such as physiological changes, pathological injuries, and environmental changes. Its essence is the brain’s self-regulatory mechanism to cope with chronic stress, injuries, and other changes through structural and functional changes (Kourosh-Arami et al., 2021). Neuronal plasticity is an important foundation for the recovery of structure and function in the central nervous system after lesions (Chernikova et al., 2013). Neuronal plasticity can be divided into structural plasticity and functional plasticity. Structural plasticity involves functional brain area anatomical and morphological changes such as neurogenesis, synaptogenesis, angiogenesis, axonal or dendritic sprouting, etc. Functional plasticity refers to the changes in neural network function and the formation of new neural pathways (Dan, 2019). There was a belief that neuroplasticity is stronger during early individual development and gradually diminishes in adulthood. However, both theoretical and practical research have refuted this viewpoint. In fact, neuroplasticity exhibits stage-specific and modular differences. During human development, neuroplasticity is strongest; in adulthood, certain brain regions such as the sensory cortex experience a decrease in plasticity, but areas like the motor cortex, prefrontal cortex, and hippocampus maintain a high level of plasticity (Karni et al., 1995; Sarı and Lunghi, 2023).

As an important discovery in neuroscience, neuroplasticity is the fundamental basis for the survival and repair of the nervous system after injury. Influencing neuroplasticity through certain interventions to promote injury recovery and pathological compensation has become an important treatment concept. As mentioned earlier, certain brain regions always have a high level of neuroplasticity. Therefore, inducing plasticity changes through certain stimuli such as exercise, cognitive training, and environmental changes may have unexpected therapeutic effects (Innocenti, 2022). In recent years, treatment techniques based on the theory of neuroplasticity have emerged continuously. Among them, non-invasive brain stimulation techniques that target brain functional areas and regulate interhemispheric function are typical representatives (Braun and Wittenberg, 2021). Furthermore, as research deepens, exercise, as a non-pharmacological therapy, has also shown great potential in regulating cerebral vascular function, inducing neuroplasticity, and inhibiting neuroinflammation (Reddy et al., 2023). Exercise can have beneficial effects on brain structure and function. Exercise can regulate neural activity, promote the expression of neurotrophic factors, and facilitate signal transduction. The increased release of neurotrophic factors further promotes neuronal production, proliferation, and differentiation; increases synaptic formation, enhances neural conduction efficiency, and improves functional neural activity; stimulates angiogenesis, increases cerebral perfusion, and achieves beneficial changes in neural structure and function. Exercise also promotes positive changes in the structural morphology of the brain, activates relevant functional brain regions, and promotes adaptive behavioral changes (Zhao et al., 2020). In addition, exercise can increase cortico-spinal excitability and have a positive impact on functional connectivity, gray matter density, and white matter microstructure in the motor area (Nicolini et al., 2021). The theory of exercise-induced neuroplasticity is being mentioned and studied by an increasing number of experts and scholars. Recent clinical practice has shown that CLBP is characterized by a long course, abnormal nociceptive processing mechanisms, decreased motor control function, and secondary changes in related functional brain areas, which may be important neuroplasticity changes (Brumagne et al., 2019). Induced neuroplasticity changes may be the key to the rehabilitation treatment of CLBP and its various complications.

## 3 Molecular mechanisms of exercise-induced neuroplasticity

Brain-derived neurotrophic factor (BDNF) is a key protein involved in neuroplasticity events after exercise (Kaagman et al., 2024). BDNF is a widely studied neurotrophic factor that plays an important regulatory role in neuronal function and brain health (Jaberi and Fahnestock, 2023). As the most widely distributed neurotrophic factor, BDNF can promote neuroplasticity throughout the lifespan (Voigt et al., 2024). BDNF can promote the differentiation of neural stem cells into astrocytes and oligodendrocytes, making it a key factor in neuroglial development. BDNF plays an important role in the process of neurogenesis, neuron survival, proliferation, and differentiation. The main mechanism is that mature BDNF (mBDNF) can bind to the tropomyosin receptor kinase B (TrkB) receptor, activate signaling pathways such as mitogen-activated protein kinase (MAPK), phospholipase C-γ (PLC-γ), phosphatidylinositol 3-kinase (PI3K)/Akt enzyme (Akt, also known as protein kinase B), and then activate transcription factor cyclic adenosine monophosphate (cAMP) response element-binding protein, playing roles in anti-apoptosis and synthesis of cytoskeletal proteins (Palasz et al., 2020). Neuronal generation, differentiation, and development are more active in regions such as the hippocampus, cerebellum, and cerebral cortex, where BDNF plays an important role. BDNF regulates synaptic formation and growth and promotes synaptogenesis (Binder and Scharfman, 2004), while BDNF stimulates vascular endothelial growth factor transcription, which in turn is involved in neoangiogenesis (Lin et al., 2014). In addition, BDNF exhibits neuroprotective effects under adverse conditions such as glutamate excitotoxicity, neurotoxic stimulation, and cerebral ischemia. In addition, BDNF is also a key molecule in brain learning and memory, playing an important role in cognitive function and preventing neurodegenerative changes (Decker, 2023). Some scholars have suggested that physical exercise improves brain health by promoting neurogenesis and modulating neuroplasticity and that an important molecular mechanism is the increased expression of BDNF (Cefis et al., 2023). One research has found that long-term aerobic exercise can increase the concentration of BDNF, insulin-like growth factor 1 (IGF-1), vascular endothelial growth factor (VEGF), and other factors. BDNF promotes neuroplasticity throughout the lifespan, IGF-1 is crucial for brain development and normal function maintenance, and VEGF promotes neural precursor proliferation (El-Sayes et al., 2019). A systematic review and network meta-analysis study showed that high-intensity interval training also induces neuroplastic changes in the brain by increasing BDNF levels (Rodríguez-Gutiérrez et al., 2024).

**Figure 1:**
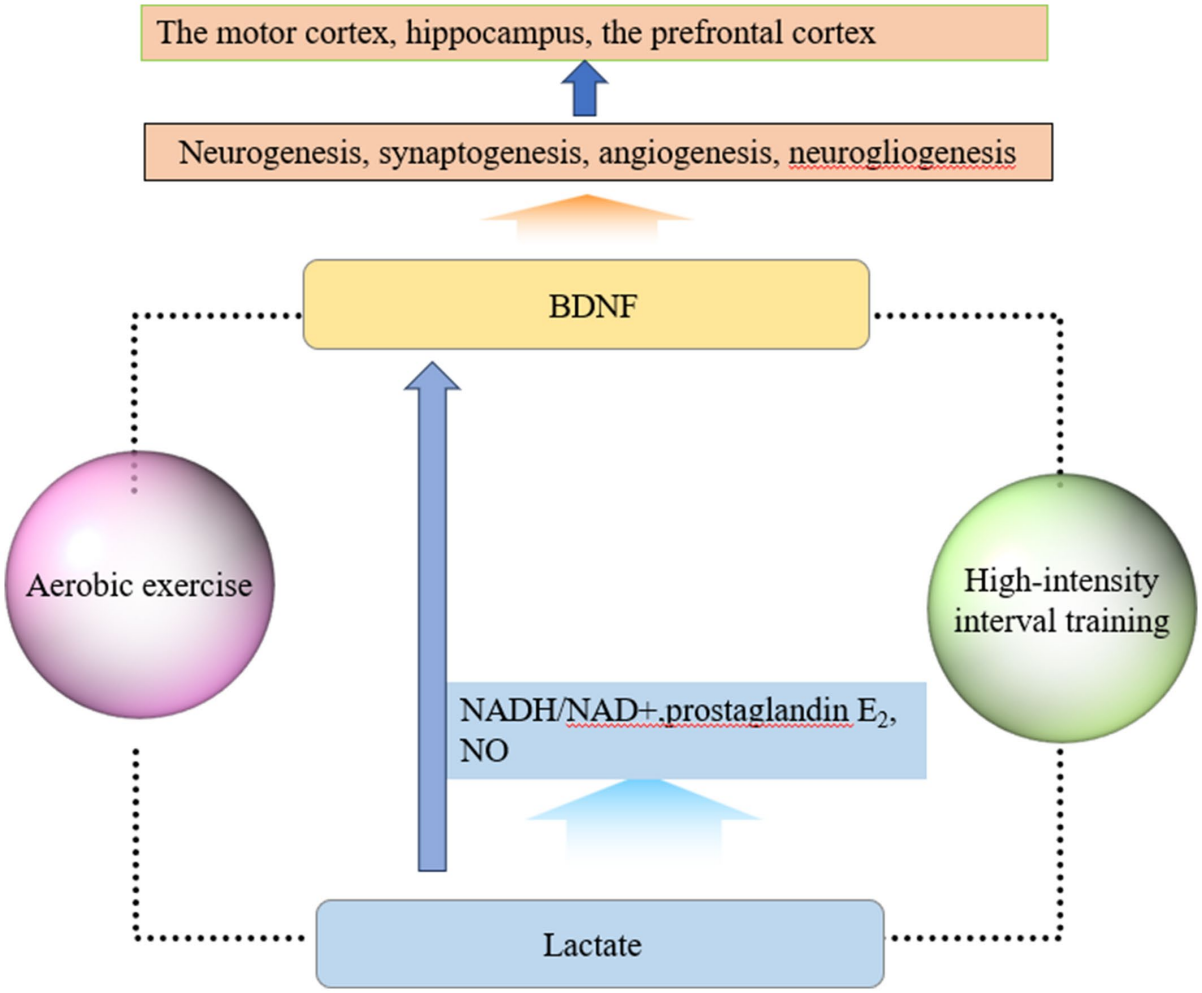
Molecular mechanisms of exercise-induced neuroplasticity. (1) Exercise elevates the level of BDNF, which plays important roles in neurogenesis, synaptogenesis, angiogenesis, and neurogliogenesis. BDNF can promote neuroplasticity changes, especially in brain regions such as the motor cortex, hippocampus, and prefrontal cortex. (2) Muscles can produce lactate, which enters the bloodstream and crosses the blood–brain barrier through monocarboxylate transporters during exercise. As lactate enters the brain, the NADH/NAD+ ratio, prostaglandin E2, and NO production increase, which can cause cerebral vasodilation and improve cerebral blood circulation. Lactate can also induce neurovascular coupling, increase BDNF expression and synthesis, and mediate neuroplasticity. Based on current research findings, exercise in this figure refers to two forms of exercise: aerobic exercise and high-intensity interval exercise.

Another key signaling molecule that may be involved in exercise-induced neuroplasticity is lactate (Lei et al., 2024). In recent years, the role of lactate in brain development and functional maintenance, synaptic plasticity, and angiogenesis has attracted the attention of scholars (Wu et al., 2023). During exercise, muscles can produce lactate, which enters the bloodstream and crosses the blood–brain barrier through monocarboxylate transporters. Another source of lactate in the brain is glycolysis within astrocytes. Exercise causes an increase in neuronal metabolic demand, and a portion of the glycogen stored in astrocytes is degraded to lactate to maintain neuronal function (Akter et al., 2023). As the level of lactate in the brain increases sharply, the NADH/NAD+ ratio increases, leading to an increase in prostaglandin E2 and nitric oxide production, causing cerebral vasodilation and improving cerebral blood circulation. In addition, lactate can also induce neurovascular coupling through certain mechanisms, increase BDNF expression and synthesis, and mediate neuroplasticity. Research has shown that the increase in lactate levels after high-intensity exercise is related to the increase in BDNF levels (Müller et al., 2020). Lactate, as a signaling molecule, improves cerebral blood flow and increases the release of BDNF, which is a possible important mechanism for exercise-induced neuroplasticity. High-intensity interval training can increase lactate levels and improve cerebral vascular function through related signaling pathways, activating neuroplasticity (Huang et al., 2021). A Systematic Review showed that high-intensity interval training increased plasma concentrations of neuroplasticity markers such as lactate, BDNF, and VEGF in the brains of stroke patients and promoted functional recovery (Montero-Almagro et al., 2024). Another animal study showed that high-intensity intermittent training promoted hippocampal mitochondrial function and ameliorated neuronal synaptic dysfunction via the lactate receptor (GPR81)-ERK1/2 pathway, in which lactate plays a mediating role (Shang et al., 2023). In addition, aerobic exercise may also improve cortical inhibition function by elevating lactate levels, and one clinical study found that patients who produced higher lactate during aerobic exercise achieved faster response inhibition. In conclusion, lactate plays a role in the regulation of neuronal excitability and the induction of synaptic plasticity, In the future, the specific mechanisms of lactate in neural network regulation need to be further investigated (Griego and Galván, 2023). In summary, the molecular mechanisms of exercise-induced neuroplasticity are shown in Figure 1.

## 4 A new perspective on exercise therapy for chronic low back pain: targeting neuroplasticity

### 4.1 Modulating excitability of the cerebral cortex and conduction pathways

Cortical excitation and inhibition (E/I) balance is one of the important mechanisms for maintaining its internal homeostasis and normal function. Studies have shown that patients with CLBP have disrupted E/I balance in the motor and prefrontal cortices (Sarı and Lunghi, 2023), and the resulting excitability changes in the related cortices and related transmission pathways may be one of the important reasons for the persistence of pain (Parker et al., 2016; Schabrun et al., 2017). Cortical excitability includes two forms: short-interval intracortical inhibition (SICI) and short-interval intracortical facilitation (SICF). Studies have found that individuals with CLBP have lower short-interval intracortical facilitation (SICF) and cortical-spinal excitability than normal individuals, which may be related to defects in cortical glutamate regulation mechanisms (Corti et al., 2022). Therefore, altering cortical excitability may be the key to improving clinical symptoms such as pain, functional impairment, cognitive and behavioral changes, depression, and fatigue in chronic pain patients (Mhalla et al., 2010). The decreased stability of the lumbar spine caused by dysfunction of the core muscle groups such as the trunk, abdomen, and paraspinal muscles is one of the important factors contributing to the persistent pain and functional impairment in patients with CLBP. The motor cortex, especially the primary motor cortex (M1), plays an important role in motor control, and changes in its excitability can lead to motor control impairments, thereby affecting lumbar spine stability. Motor control training can induce protein synthesis changes, synaptic plasticity, and functional reorganization in M1, and affect the local release of GABA in the M1 region, increasing the excitability of the cortico-spinal pathway and enhancing the efficacy of neural synapses. By influencing the plasticity of M1, it can promote the restoration of the activation timing of the core muscle groups and maintain this motor control mechanism in functional tasks through learning, thereby enhancing lumbar spine stability and achieving therapeutic effects (Massé-Alarie and Schneider, 2016). Another study found that aerobic exercise can reduce cortical inhibition and/or increase cortical facilitation, and the repetitive activation of neural networks can endogenously influence neural plasticity (Clos et al., 2021).

### 4.2 Promoting neurogenesis and synaptic plasticity in the hippocampus

The hippocampus is an important region for neurogenesis and is associated with the perception, cognition, and emotional processing of chronic pain. Abnormal changes in the hippocampus have been found in animal models and humans with chronic pain. Animal studies have found that glutamate levels increase in the hippocampus during chronic pain, and excessive excitability in the hippocampus can alter synaptic plasticity in the periaqueductal gray, leading to the chronification of pain (Bilbao et al., 2018). Further clinical research has found inconsistent gender differences in chronic pain, with higher incidence and more complications in females, possibly related to anatomical changes in the left hippocampus, leading to functional connectivity and emotional regulation disorders (Reckziegel et al., 2021). Basic research shows that neuronal apoptosis and synaptic changes in the hippocampus can induce depression and affect learning and memory. Chronic pain patients may have reduced hippocampal volume, manifested as anxiety, depression, and learning and memory deficits, which may be due to decreased neurogenesis and changes in synaptic plasticity (Mutso et al., 2012; Barroso et al., 2021). The hippocampus is located in the medial temporal lobe, and its dentate gyrus structure is an important area for neurogenesis, playing a crucial role in spatial learning and memory processes. Chronic stress or pain can increase glucocorticoid and excitatory amino acid levels, leading to dysfunction of the hypothalamic–pituitary–adrenal axis, while neurogenesis in the dentate gyrus can also be inhibited, resulting in structural atrophy and depression (McEwen, 2001). An epidemiological study showed that the prevalence of anxiety and depression in CLBP patients was 19 and 25%, respectively (Stubbs et al., 2016). The synaptic plasticity changes in the hippocampus are an important cause of depression. Synaptic plasticity refers to activity-dependent changes in synaptic strength or transmission efficiency. The most extensively studied forms of synaptic plasticity are long-term potentiation (LTP) and long-term depression (LTD) of synaptic transmission (Andrade-Talavera et al., 2023). BDNF is involved in the regulation of hippocampal synaptic plasticity (Kowiański et al., 2018). BDNF has two hydrolytic forms, proBDNF and mBDNF, which activate different receptors and generate opposite biological effects through corresponding molecular signaling pathways. proBDNF can bind to the p75 neurotrophin receptor, inducing neuronal apoptosis, LTP, and amyloid-like degeneration. On the other hand, mBDNF binds to TrkB, producing protective effects, LTP, regulating synaptic plasticity, promoting neuronal development, proliferation, and dendritic and axonal growth (Sun et al., 2023; Wang et al., 2024). The ratio of proBDNF to mBDNF varies with different brain regions and developmental stages, and can also be altered by pathological factors. Based on the important role of proBDNF/mBDNF balance in regulating neuronal and synaptic function, it is believed that the decreased BDNF expression in the hippocampus and prefrontal cortex, as well as the resulting changes in synaptic plasticity, are the causes of depression (Pisani et al., 2023). Synaptic plasticity is involved in brain network reconstruction and clinical recovery after brain injury (Bassi et al., 2019; Bettio et al., 2019). Different forms of exercise, such as running, light activity, and aerobic exercise, can regulate synaptic plasticity, improve cognition, and enhance spatial learning and memory (Brockett et al., 2015; Khoury et al., 2023). Research shows that during active movement in mice, the liver can release *β*-hydroxybutyrate, which inhibits the deacetylation of histone acetyltransferase and increases BDNF levels in the hippocampus, thereby promoting synaptic plasticity (Byun et al., 2023). Aerobic exercise can increase the number of synapses and dendritic density in the medial prefrontal cortex and hippocampus, thereby improving brain functional connectivity (Erickson et al., 2011).

### 4.3 Targeting the prefrontal cortex to improve cognitive behavioral disorders

Chronic pain patients often exhibit pain catastrophizing, fear of movement, and executive attention deficits (Marshall et al., 2017). Changes in cognitive, emotional, and behavioral aspects in patients with CLBP interact with structural and functional impairments, and brain changes may be a key factor in this interaction. Under the continuous stimulation of pain, the body’s self-regulation mechanism for dealing with negative thoughts is abnormal, leading to abnormal emotional and behavioral manifestations such as pain catastrophizing and fear of movement (Bunzli et al., 2017). From the perspective of the modern biopsychosocial medical model, the continuous stimulation of chronic pain can lead to maladaptation of the body. The patient’s attention is difficult to shift from the pain, and feelings of helplessness and anxiety during the pain experience can amplify its impact on the body, resulting in pain catastrophizing thoughts. Excessive fear of the pain-inducing stimuli and irrational fear of potential damage can cause patients to develop fear of movement. Both are adverse cognitive and emotional responses related to pain (Bordeleau et al., 2022). Excessive reactions to pain can also lead to impairments in memory, executive function, attention, and other cognitive domains (Berryman et al., 2013).

Changes in cognitive behavior in patients with CLBP are related to cortical reorganization, which is an important clinical marker for poor prognosis and disease progression (Smeets et al., 2009). Studies have shown that about one-third of chronic pain patients have cognitive impairments in learning, memory, attention, and decision-making (Moriarty et al., 2011). The prefrontal cortex (PFC) is an important region for cognitive function, involved in complex cognitive behaviors such as memory, social interaction, and behavioral decision-making. It also plays an important role in pain perception and emotion regulation as a key component of the midbrain-limbic system (Arnsten, 2015). Research has found that the PFC region in chronic pain patients has cortical thinning (Yang et al., 2020). Further research has revealed that the dorsolateral prefrontal cortex (DLPFC) may be responsible for the appearance of symptoms such as catastrophizing thoughts, fear of movement, and cognitive deficits in chronic pain patients (De Ridder et al., 2021). DLPFC is an important functional area involved in the processing of pain, cognition, and emotion. When there is sustained nociceptive stimulation or an increased demand for DLPFC function, the efficiency of cognitive functions decreases, leading to abnormal regulation of DLPFC (Seminowicz and Moayedi, 2017). A study showed that patients with CLBP had reduced cortical thickness in the left DLPFC compared to healthy subjects. After effective treatment, the cortical thickness in the relevant area increased, and beneficial changes in the connectivity between the PFC and other brain regions were observed. The increase in DLPFC thickness and improvement in functional activity were associated with the improvement of pain and cognitive impairments (Seminowicz et al., 2011), indicating that the changes in brain function and structure in CLBP patients are reversible. Clinical studies have also found that aerobic exercise can increase the volume and functional activity of the prefrontal and temporal cortex hippocampal regions. In addition, exercise improves cognition, and the increase in levels of products such as BDNF during exercise may promote long-term synaptic potentiation, which could be a potential mechanism (Vints et al., 2022).

### 4.4 Delaying brain degeneration caused by aging and low back pain

As the aging process progresses, the elderly population experiences insufficient brain perfusion and microcirculation disorders, resulting in reduced gray and white matter volume. This leads to impaired motor control and function, gait disturbances, increased risk of falls, and cognitive decline. In addition, external stimuli such as stress and lack of physical activity can lead to decreased expression of BDNF and weakened or even death of neurons, which is also a significant cause of brain dysfunction (Dadkhah et al., 2024). Research has found that plasma BDNF levels decline with age (Lommatzsch et al., 2005), and BDNF is crucial for maintaining brain function. Ensuring the maintenance of BDNF is of utmost importance. CLBP can have many adverse effects on the body, and the incidence increases with age. A study showed that the proportion of CLBP in the population aged 65 and above is 12% (Arnstein, 2010). Elderly patients with CLBP are affected by both back pain and natural aging. Research has found that CLBP presents neurodegenerative characteristics such as neuronal apoptosis and decreased gray matter in the brain as the disease progresses (Mosabbir, 2022). Furthermore, CLBP patients have abnormal brain metabolism, which activates neuroglial cells that play a role in immune surveillance, leading to increased release of pro-inflammatory and chemotactic factors, resulting in the appearance of neuroinflammation (Ji et al., 2018; Albrecht et al., 2021; Li et al., 2021). Research has found that chronic pain patients have changes in brain metabolism, which can lead to degeneration of the thalamus and prefrontal cortex. Brain regions such as the hippocampus, basal ganglia, and amygdala can also be affected, and these regions are closely related to memory, movement, and emotion processing (Mosabbir, 2022). Exercise can effectively address the dual impact of aging and lower back pain. Exercise can increase the expression of vascular endothelial growth factor, induce vascular generation in certain brain regions, improve brain blood supply, delay brain volume reduction, and promote motor learning or relearning through stimulating neuroplasticity (Nishijima et al., 2016). Exercise has non-invasive characteristics and has antioxidant and stress-reducing effects, which can reduce neuroinflammation and protect neurons (Rezaee et al., 2022). Studies have shown that long-term aerobic exercise can increase cortical volume and hippocampal volume, enhance brain connectivity and activation, maintain white matter integrity, and reduce the risk of cognitive decline. For elderly and frail patients, centrifugal motion requires relatively low cardiovascular and pulmonary function, and can induce neuroplasticity improvement in overall function through hemodynamic changes and repeated activation of the neural network and other endogenous mechanisms (Clos et al., 2021).

## 5 Summary and outlook

**Figure 2:**
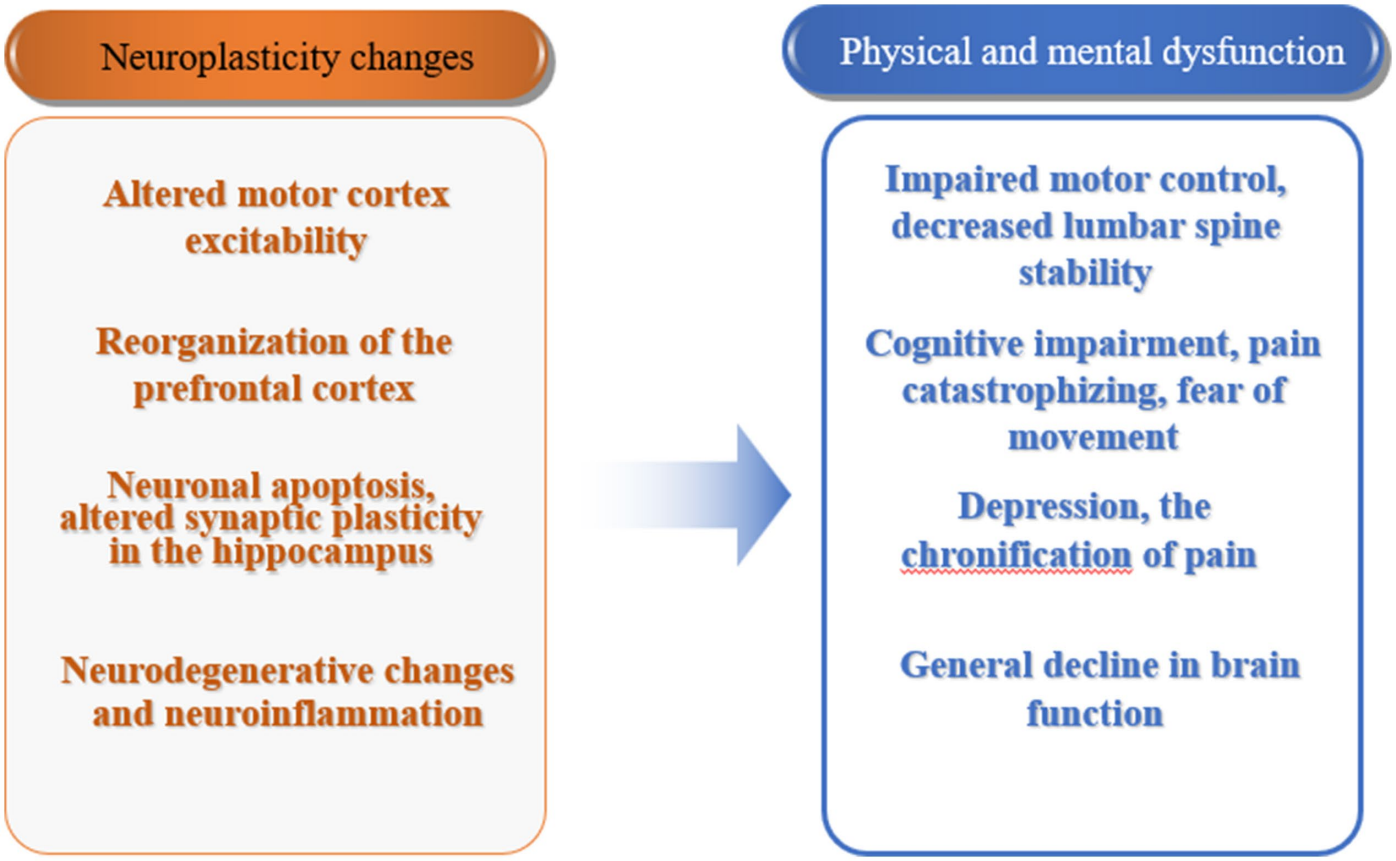
Neuroplasticity changes and physical and mental dysfunction in patients with chronic low back pain.

Neuroplasticity changes in patients with CLBP are an important mechanism leading to their physical and mental functional impairments (Figure 2). Under physiological conditions, neuroplasticity mechanisms ensure the normal development of life activities. Under the influence of pathological factors, changes in the central nervous system may have adverse effects on the body. Therefore, reversing the changes in brain structure and network activation or reorganization associated with pain behavior through certain means has become a new treatment approach (Medrano-Escalada et al., 2022). Currently, there are two ways to achieve this: one is from the central to the peripheral, including motor imagery, cognitive behavioral therapy, non-invasive brain stimulation techniques, etc., especially the combination of non-invasive brain stimulation techniques and pain neuroscience education (PNE) has become an important direction for future development. The other way is from the peripheral to the central, achieved through exercise, manual therapy, and stimulation of peripheral physical factors. Obviously, exercise induces neuroplastic changes through the latter pathway, thereby achieving corresponding rehabilitation effects. In recent years, mind–body exercise has been widely carried out, which implies the rehabilitation treatment concept of central-peripheral. Currently, traditional Chinese exercises such as qigong, yoga, and Pilates, which represent mind–body exercise, have been applied in the treatment of low back pain in clinical practice. Mind–body exercise is based on attentional focus, using mindfulness, meditation, guided imagery, etc., combined with gentle movements and breath control to enhance mind–body coordination and awareness, increase vitality, promote energy flow and release of endogenous neurohormones, activate self-regulation ability, and induce neuroplastic changes (Zou et al., 2017). In the future, the integration of physical and cognitive behavioral regulation in exercise may play a greater role in the rehabilitation treatment of CLBP, and its mechanism deserves further research and exploration.

## Data availability statement

The original contributions presented in the study are included in the article/supplementary material, further inquiries can be directed to the corresponding author.

## Author contributions

JZ: Writing – original draft. SH: Writing – review & editing.
